# Mechanical Properties and Crystallinity of Specific PLA/Cellulose Composites by Surface Modification of Nanofibrillated Cellulose

**DOI:** 10.3390/polym16172474

**Published:** 2024-08-30

**Authors:** Hongzhe Chu, Zeyan Chen, Yongli Chen, Deling Wei, Yang Liu, Hui Zhao

**Affiliations:** 1 Guangxi Key Laboratory of Clean Pulp & Papermaking and Pollution Control, School of Light Industry and Food Engineering, Guangxi University, Nanning 530004, China; 2105170333@st.gxu.edu.cn (H.C.); 2105170335@st.gxu.edu.cn (Z.C.); xiaobai@gxu.edu.cn (Y.L.); 2 School of Environmental and Life Science, Nanning Normal University, Nanning 530100, China; 18278531535@163.com

**Keywords:** nanofibrillated cellulose, polylactic acid, composites, crystallinity, mechanical properties

## Abstract

Polylactic acid (PLA) has inherent drawbacks, such as its amorphous structure, which affect its mechanical and barrier properties. The use of nanofibrillated cellulose (NFC) mixed with PLA for the production of composites has been chosen as a solution to the above problems. A PLA/NFC composite was produced by solution casting. Before use, the cellulose was modified using a silane coupling agent. The composite films were investigated via X-ray diffraction, as well as by mechanical, physical, thermal analyses and by differential scanning calorimeter. The crystallinity was four times that of pure PLA and the water vapor transmission rate decreased by 76.9% with the incorporation of 10 wt% of NFC. The tensile strength of PLA/NFC blend films increased by 98.8% with the incorporation of 5 wt% of NFC. The study demonstrates that the addition of NFC improved the properties of PLA. This provides a solid foundation for the enhancement of the performance of PLA products.

## 1. Introduction Mechanical Properties

Polylactic acid (PLA) has garnered attention due to its abundancy, broad availability, renewability, biodegradability, and strong processability [1]. Nowadays, PLA is taken as the preferred biopolymer for preparing petroleum-based plastics alternatives. From 2011 to 2020, global PLA output increased from less than 200 kt/year to about 800 kt/year [2]. However, its amorphous structure leads to poor crystallinity, mechanical properties, and barrier properties [3,4]. Among various methods, adding heterogeneous nucleating agents with high nucleation density to increase crystallization rate or crystallinity is an effective approach.

Nanofibrillated cellulose (NFC), extracted from cellulose, not only possesses all the advantages of cellulose but also exhibits nano-size effects such as nanoscale dimensions, excellent mechanical properties, large specific surface area, and high surface reactivity [5]. With the increase of NFC length, the mechanical properties first increased and then decreased [6]. In high humidity environments, the mechanical properties of cellulose decrease as the aspect ratio decreases [7]. This makes NFC an ideal choice as a reinforcing phase for composites like polylactic acid. Dissolving cellulose has become a focal point of current research. In this paper, we use non-derivatized solvents to dissolve cellulose, which achieve dissolution through physical molecular interactions. High-concentration inorganic salt solution systems are widely studied due to their green, non-toxic properties, ease of preparation, and low cost. The cellulose dissolved in a ZnCl_2_/AlCl_3_-4H_2_O solvent system has a lower degree of degradation, retaining the original properties of cellulose without damaging its excellent mechanical properties [8]. This article first dissolves NFC using an aqueous solution of ZnCl_2_ and AlCl_3_. We explore the optimal ratio of ZnCl_2_, AlCl_3_, and H_2_O for dissolution and analyze the dissolution mechanism by comparing it with original NFC. To investigate the impact of dissolution on modification, we compare and analyze the elemental, thermal, and crystallization properties of untreated NFC, directly modified NFC, and dissolved-then-modified NFC. Nanocellulose can function as a nucleating agent in the PLA matrix, and heterogeneous nucleation can promote PLA crystallization [9,10]. Additionally, the high aspect ratio of nanocellulose can extend the water vapor or gas permeation path in the PLA matrix, improving its moisture permeability [11]. Although NFC is considered a good nanofiller, it is mostly dispersed in water as a white slurry. Due to its large specific surface area and strong hydrophilic properties, as well as the presence of numerous polar hydroxyl groups on its surface, cellulose molecules can aggregate under strong hydrogen bonding during drying [12,13]. Once aggregated, NFC is difficult to disperse. Furthermore, the weak hydrogen bonds formed between the nonpolar C=O groups in the PLA molecular structure and nanocellulose result in a weak interfacial adhesion, which limits the reinforcing effect of NFC on PLA [14]. Therefore, improving the compatibility and dispersion of NFC in PLA is a significant challenge in NFC-reinforced PLA research. Currently, methods to enhance the compatibility between nanocellulose and the PLA matrix primarily include adding compatibilizers and surface modification of NFC [15,16]. The main blending methods for NFC and PLA include melt blending, solution casting, and electrospinning. Melt blending primarily involves the incorporation of cellulose nanofibers (CNF) into the thermoplastic polymer polylactic acid (PLA). This process utilizes the high shear and elevated temperatures of the machinery to achieve a uniform melt blending of CNF and PLA, followed by hot pressing. In the solution casting method, CNF is dissolved and modified, which weakens the hydrogen bonding between the fibers, thereby reducing agglomeration and enhancing dispersion within the solution before it is cast into a film. Electrospinning employs electrostatic forces to eject nanofibers from either a polymer solution or a polymer melt, producing a charged jet. This solidified jet is then deposited onto a collector, forming fibers that contribute to the creation of a composite material.

In this paper, modified NFC is mixed with PLA in dichloromethane solution, and PLA/NFC nanocomposites with varying NFC content are prepared using a casting method. The surface morphology, crystallization behavior, thermal properties, rheological properties, mechanical properties, and moisture permeability of pure PLA and PLA/NFC nanocomposites are investigated. The goal is to develop PLA/NFC composite films based on modified NFC to improve interfacial compatibility.

## 2. Materials and Methods

### 2.1. Materials

Polylactic acid (PLA) was purchased from Zhejiang Hisun Biomaterials Co., Ltd. with a molecular weight of 100,000–150,000. NFC, Softwood pulp, was purchased from Zhongshan Nanofiber New Materials Co., Ltd. Dichloromethane (DCM) and Zinc chloride (ZnCl_2_) was purchased from Tianjin Zhiyuan Chemical Reagent Co., Ltd., Tianjin, China. Aluminum chloride hexahydrate (AlCl_3_·6H_2_O) was purchased from Guangdong Guanghua Sci-tech Co., Ltd., Shantou, China.

### 2.2. Dissolution and Modification of NFC

NFC was dissolved using a mixed aqueous solution of ZnCl_2_ and AlCl_3_. An ionic solution was prepared with a ratio of Zn^2+^, Al^3+^, and H_2_O at 0.9:0.1:3. Keeping the total molar amounts of Zn^2+^ and Al^3+^ as 1 mole, 1 wt% of NFC was added to each proportion of the metal salt solution. The NFC was dissolved by stirring at ambient temperature until the solution became transparent with no residue. The corresponding ethanol solution was taken according to the amount of water used in the dissolution, so that the ratio of water and ethanol was 1:4. The pH value of the solution was adjusted to 3–4, and the resulting solution was stirred thoroughly and set aside. After the NFC was completely dissolved, we added the silanization modification solution and stirred it in an 80 °C oil bath for 12 h. Then the solution obtained was centrifuged, and the excess ethanol solution was added to the NFC for rinsing and centrifugation 3 times. After drying, the dissolution modified NFC was finally obtained, which was recorded as DMNFC.

### 2.3. Preparation of PLA/NFC Composite Films

**Figure 1:**
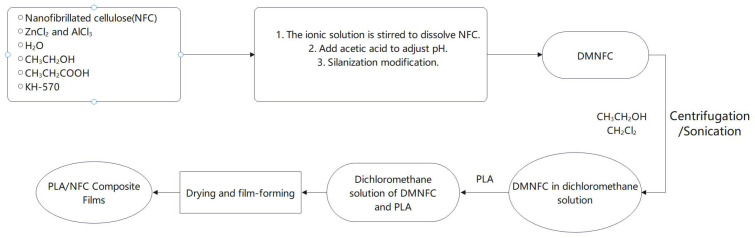
Flowchart for preparation of PLA/NFC composite films.

To disperse NFC in a dichloromethane solution for subsequent mixing with polylactic acid (PLA), ethanol was added to the aqueous dispersion of NFC to eliminate unreacted silane modifiers. The mixture was then centrifuged, and the solid material obtained after centrifugation was retained. The liquid was discarded, and this process was repeated three times to yield an NFC solution dispersed in ethanol; then dichloromethane was added to this solution, and the same centrifugation operation was repeated three times to obtain the NFC solution dispersed in dichloromethane. Finally, the solution was subjected to sonication for 30 min to further disperse it. The NFC solutions were configured with different ratios of NFC to PLA dry weight, poured into films and dried for characterization (Figure 1).

### 2.4. Characterization

#### 2.4.1. Scanning Electron Microscopy (SEM) and Energy Dispersive Spectrometer (EDS) Analysis

Surface morphologies of cellulose, modified cellulose, DMNFC, PLA, and PLA/NFC composites with different proportions were observed by SEM (SU8020, Hitachi high-tech group, Japan) and EDS (iS 50, Thermo Fisher Scientific, Waltham, MA, USA). The sample was stuck on the conductive adhesive and sprayed with gold for 30 s. The sample was observed at a canning voltage of 10 KV. An EDS area scan was performed on the sample morphology captured by SEM, targeting the elements C, O, and Si.

#### 2.4.2. X-ray Photoelectron Spectroscopy (XPS) Analysis 

The surface chemical composition of cellulose and modified cellulose fiber was determined by XPS (ESCALAB 250XI+, Thermo Fisher Scientific, Waltham, MA, USA). The test conditions were as follows: voltage 12 kV, current 6 mA; full-spectrum scanning fluence energy of 150 eV with a step of 1 eV; and narrow-spectrum scanning fluence energy of 50 eV with a step of 0.1 eV.

#### 2.4.3. X-ray Diffraction (XRD) Analysis

The crystallinity of cellulose, modified cellulose fiber, PLA and different proportions of PLA/NFC composites were determined by XRD (ESCALAB 250XI+, Thermo Fisher Scientific, Waltham, MA, USA). The tests were recorded using Cu-Kα rays at a wavelength of λ = 0.154 nm and the samples were scanned at a voltage of 40 kV and a current of 30 mA. The scanning diffraction angle 2θ ranged from 5° to 50° and the scanning rate was 6°/min.

#### 2.4.4. Differential Scanning Calorimeter (DSC) Analysis

The PLA and PLA/NFC composites with different ratios were dried in an oven at 60 °C for 6 h. The test conditions in DSC (ESCALAB 250XI+, Thermo Fisher Scientific, Waltham, MA, USA) were as follows: (1) The temperature was raised from room temperature to 180 °C at a heating rate of 10 °C/min and held at 180 °C for 5 min; (2) the temperature was cooled from 180 °C to room temperature at a rate of 2 °C/min; (3) the temperature was again raised from room temperature to 180 °C, but this time at a heating rate of 2 °C/min. The entire heating and cooling process was conducted under N_2_ with a gas flow rate of 40 mL/min. The crystallinity (Χc) of the materials was then calculated using the second heating curve obtained from step 3, as shown in Equation (1).

(1) $$X_{c}\left(\%\right)=\frac{\Delta H_{m}-\Delta H_{cc}}{\omega \Delta H_{m}^{0}}\times 100\%$$

where ΔH_cc_ is the cold crystallization enthalpy of the PLA component obtained from the test, in J/g; ΔH_m_ is the melting enthalpy of the PLA component obtained from the test, in J/g; ∆H^0^_m_ is the melting enthalpy of pure PLA with 100% crystallinity, which is a fixed value of 93.6 J/g; ref. [17] and ω is the content of PLA in the composite material.

#### 2.4.5. Thermal Gravimetry-Infrared Spectrum Analysis (TG-IR)

The NFC, MNFC, DMNFC and PLA/NFC composites were tested using a combined testing system TG-IR (TGA55, TA, Everett, MA, USA). The test conditions were as follows: the temperature increase range was from 30 °C to 800 °C, and the temperature increase rate was 10 °C/min. The whole temperature increase process was carried out in N_2_ with an air flow rate of 20 mL/min.

#### 2.4.6. Rheological Performance Test

Rheological tests on PLA and PLA/NFC composites were conducted using the German HAAKE MARS4 Modular Rheometer Workstation. A parallel plate with a diameter of 20 mm and equipped with a rotational rheometer was used. After zeroing the gap between the test rotor and the test bed, the gap was set to 1 mm. The tests were performed at a temperature of 180 °C, utilizing a shear oscillation mode with an angular velocity scan range from 0.01 to 100 rad/s and a strain amplitude of 0.05. The changes in viscosity (η*), storage modulus (G′), and loss modulus (G″) as a function of angular velocity (ω) were recorded.

#### 2.4.7. Mechanics Performance Testing

The mechanical properties of PLA and PLA/NFC composites of different proportions were tested by a universal tensile machine (Instron 3367, Instron Co., Norfolk, MA, USA). According to the GB/T 1040.2-2006 standard test, the samples were cut into a rectangle of 100 mm × 10 mm, and the stretching speed was 5 mm/min. At least five samples of each composite were tested.

#### 2.4.8. Moisture Permeability Test

The moisture permeability of the material is evaluated by water vapor transmission rate (WVTR). According to GB/T 1037-1988, the WVTR of the samples was tested by the weighing method in the water vapor transmission rate tester (PERMATRAN-W3/61, MOCON, Minneapolis, MN, USA). The samples were dried in an oven at 60 °C for 6 h. For each sample to be tested, three round parallel samples with a diameter of 38 mm were taken using a standard sampler at a temperature of 23 ± 2 °C and a relative humidity of 90 ± 2%. At the end of the test, the values obtained were averaged to obtain the final WVTR.

## 3. Results and Discussion

### 3.1. Analysis of Physicochemical Properties of Modified Cellulose

#### 3.1.1. SEM Analysis

**Figure 2:**
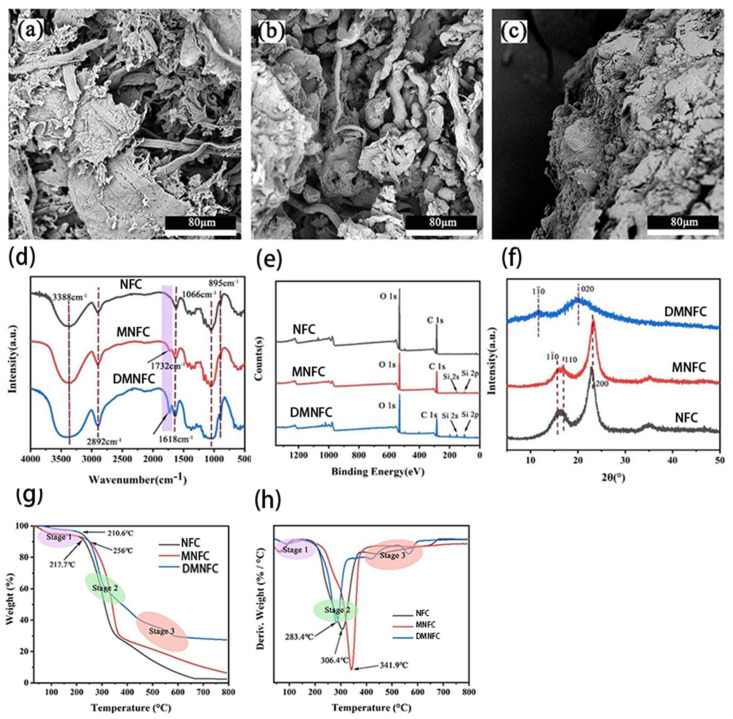
Surface morphologies of (**a**) NFC, (**b**) MNFC, and (**c**) DMNFC; (**d**) FTIR spectra of NFC, MNFC and DMNFC; (**e**) XPS spectra of NFC, MNFC, and DMNFC; (**f**) XRD patterns of NFC, MNFC, and DMNFC; (**g**) TGA of NFC, MNFC and DMNFC; and (**h**) DTG of NFC, MNFC and DMNFC.

To investigate the morphological changes of cellulose after extraction and modification, SEM images of NFC, MNFC, and DMNFC are shown in Figure 2a–c. The large number of hydroxyl groups on the surface of NFC makes it easy to be entangled, thus showing a flat ribbon structure. In Figure 2b. the spacing between modified cellulose fibers is increased. The hydroxyl groups on the NFC after the modification are partially reacted, and the bonding force is weakened. In Figure 2c, the morphology of the dissolved fibers by ions solution uniformly disappeared, and a multilamellar structure appeared, which indicates that the metal ions Zn^2+^ and Al^3+^ play a strong destructive effect on the hydrogen bonding between the NFCs.

#### 3.1.2. FTIR Analysis

The FTIR spectra of cellulose and modified cellulose are shown in Figure 2d. It can be seen that both MNFC and DMNFC show the characteristic peaks of cellulose, which are the O-H absorption peaks at 3600–3100 cm^−1^, the C-H stretching vibration peaks at 2892 cm^−1^, the O-H bending vibration peaks of adsorbed water at 1618 cm^−1^, the C-O at 1066 cm^−1^, and the -CH_2_ and C-H bending vibration peaks of the D-glucose group of cellulose at 895 cm. However, it is clear from the figure that there is one more peak of MNFC and DMNFC at 1732 cm^−1^ wavelength, which is the C=O bond present on the silane coupling agent KH-570, and it can be seen that the height of the peak of DMNFC is larger than that of MNFC, which also explains, to a certain extent, that the dissolution of the process plays a facilitating role in the modification. Among these, the absorption peaks of Si-O-C and Si-O-Si in KH-570 overlap with the cellulose C-O-C at the 1000–1200 cm^−1^ vibrational bands, which prevents them from being readily displayed in the FTIR spectra.

#### 3.1.3. XPS Analysis

XPS was utilized to investigate the changes in chemical states, electronic state changes, and element composition of the surface before and after modification. As shown in Figure 2e, the main elements identified were C and O, with electron binding energies of 286.2 eV and 532.5 eV, respectively. For the modified cellulose, two additional Si peaks (2p and 2 s) were attributed to the silicon atom from KH570. The content of Si in NFC is about 2 times more than the original amount of modification This is because the role of the hydrogen bonding between NFC makes the NFC and NFC, or NFC itself, become entangled together. The direct modification of NFC will make the modifier require only a small amount of contact reaction with the -OH bond on the NFC to achieve the purpose of modification; however, after the dissolution of this step, some of the hydrogen bonding will be destroyed, meaning that NFC on more sites can be provided for the modifier reaction, and that the modified NFC can be used for the reaction of the modifier. This means there are more sites on NFC available for the modifier to react with, that the modification effect of dissolved NFC is therefore better, and that the Si content in NFC is increased.

#### 3.1.4. XRD Analysis of Modified Cellulose

The crystalline structure of both cellulose, modified cellulose and DMNFC was characterized by XRD, as shown in Figure 2f. The diffraction peaks at 2θ = 15.9°, 17.1°and 23.2° corresponded to the characteristic (1$\bar{1}$0), (110) and (200) crystal planes of cellulose, respectively. The direct modification of NFC does not affect its crystal structure. On the other hand, the crystallographic shape of the regenerated NFC after dissolution modification was significantly changed, with obvious crystallographic peaks at 2θ = 11.6° and 20.3°, which belong to (1$\bar{1}$0) and (020), respectively, which is consistent with the cellulose type II diffraction crystal plane. This indicates that the process of dissolving these metal ions will extend into the crystalline region of nanocellulose, destroying the original hydrogen bonding structure and leading to a change in its crystalline shape. This can also indicate that dissolution has the effect of unraveling the cellulose chains that are entangled together due to hydrogen bonding, which is consistent with the FTIR test results.

#### 3.1.5. TGA and DTG Analysis

In Figure 2g, the untreated raw material NFC started to decompose in large quantities at 217.7 °C, and the T_0_ of MNFC and DMNFC were 256 °C and 210.6 °C, respectively. The onset of the decomposition temperature of MNFC increased compared with that of NFC, while the onset of the decomposition temperature of DMNFC decreased. The DTG curves also show that the T_max_ followed the same pattern in Figure 2h. This is because the introduction of silane groups into NFC modified by KH-570 hinders the movement of NFC long chains to a certain extent, which leads to an increase in the energy required for macromolecular rupture, resulting in a delay in the decomposition of NFC and an increase in thermal stability. Although Si was introduced, the effect of hydrogen bonding reduction and catalysis by metal ions was greater than the effect of Si, the result was a decrease in the T_0_ of DMNFC. After the second stage, cellulose forms amorphous carbon and the third stage is mainly the calcination of the cleavage products. Finally, we can see that the residual amount of both MNFC and DMNFC at 800 °C is higher than that of untreated NFC, while the residual amount of DMNFC is higher than that of MNFC. This is due to the presence of Si in modified NFC, so the residual amount is elevated compared with that of the untreated NFC. The Si content of DMNFC is 2.64 times higher than that in MNFC, and the presence of residual metal ions in DMNFC leads to a higher residual amount at 800 °C than MNFC.

### 3.2. Characterization of Composites

#### 3.2.1. Morphological Characteristics Analysis

**Figure 3:**
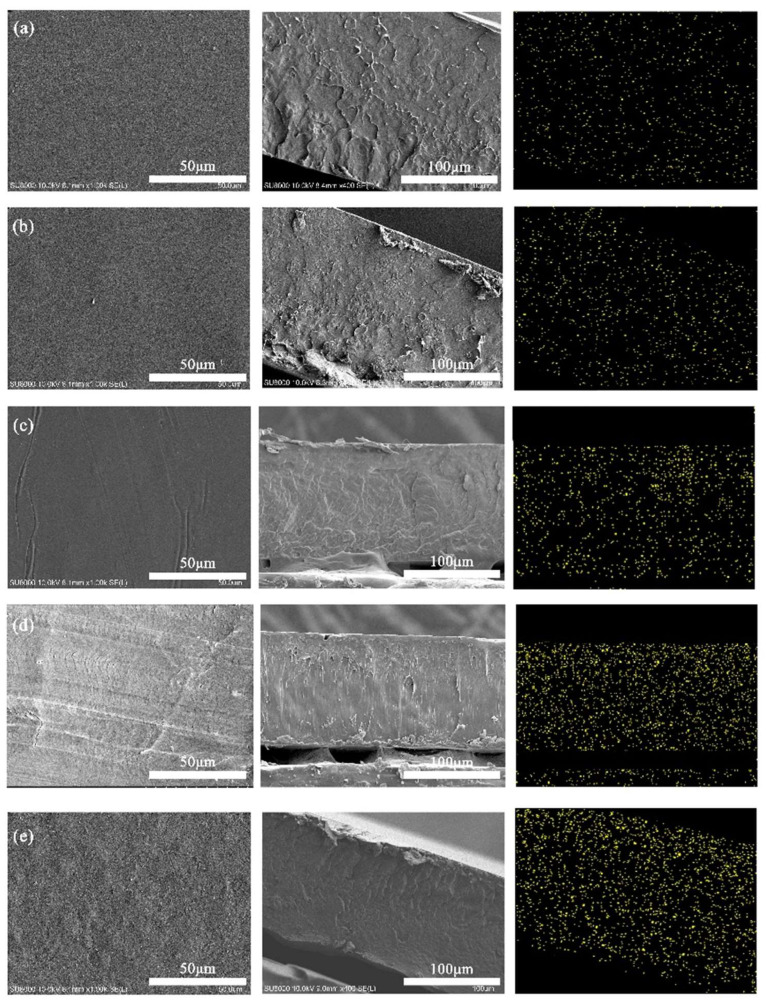
Surface morphology, cross-section morphology and EDS scan of Si elements of PLA/NFC composites (from left to right). The addition of NFC: (**a**) 1 wt%, (**b**) 3 wt%, (**c**) 5 wt%, (**d**) 10 wt% and (**e**) 15 wt%.

Tests on the surface and fracture morphology of NFC/PLA composites revealed smooth surfaces with no large NFC aggregations at 1–10 wt% NFC additions (Figure 3). However, at 15 wt%, the surface roughened, showing visible NFC aggregations. EDS scans using the Si element in modified NFC indicated regular surfaces at 1–10 wt% additions, especially smooth and dense at 10 wt%. Irregular patterns at this level suggested stronger interfacial bonding due to an intertwined NFC-PLA network, requiring higher fracture energy. Below 10 wt%, Si distribution on the fracture surface was uniform, indicating good NFC-PLA compatibility. At 15 wt%, prominent protruding patterns and rough surfaces were observed, with Si sedimentation at the bottom, due to NFC aggregation caused by hydrogen bonding, leading to material brittleness and fracture.

#### 3.2.2. XRD Analysis of Composites

**Figure 4:**
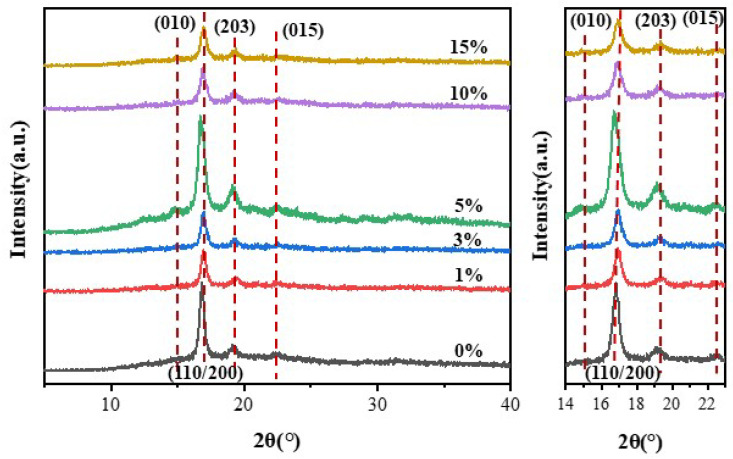
XRD curves of PLA and PLA/NFC composites with different mixed proportions.

XRD tests were conducted on the samples to investigate the effect of NFC addition on the crystallization behavior of PLA, and the results are shown in Figure 4. As can be seen from the figure, pure PLA material exhibits three diffraction peaks at 2θ = 17.2°, 19.6°, and 22.9°, which correspond to the (110/200), (203), and (015) crystal plane [18]. The coexistence of α-type and α′-type crystals is a typical characteristic diffraction peak of PLA [19]. However, the diffraction peak of the (010) crystal plane does not appear in pure PLA, indicating a low crystallinity and an amorphous structure [20]. Interestingly, upon the addition of even just 1% NFC, a diffraction peak appears at 2θ = 14.9°, corresponding to the (010) crystal plane, suggesting that NFC can promote the crystallization of PLA [21]. Upon comparing the diffraction peaks, it is observed that the addition of NFC does not change the crystal form of the composite material, as four typical PLA diffraction peaks are still present. However, as the amount of NFC added gradually increases, the peak intensities of the four diffraction peaks show a trend of increasing first and then decreasing. The peak intensities reach their highest when the NFC content is 10 wt%. This is because NFC acts as a nucleating agent in the material, forming crystal growth points in PLA, thereby improving the crystallization efficiency and crystallinity of PLA. This also indicates that NFC can be evenly dispersed in PLA, verifying the modification effect of NFC. When the NFC content exceeds 10 wt%, the peak intensities decrease. This is because excessive NFC content can lead to agglomeration in PLA, hindering the movement of PLA molecular chains and resulting in a decrease in crystallization rate, thus failing to function as a nucleating agent.

#### 3.2.3. Crystallinity and Melt Behavior Analysis

**Figure 5:**
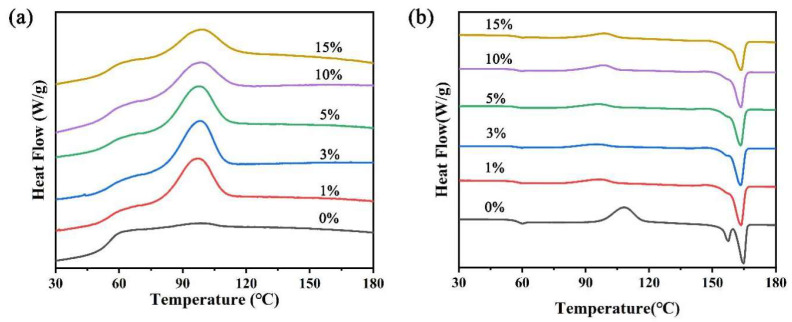
DSC thermal analysis diagram of PLA and PLA/NFC composites of different mixed proportions. (**a**) Cooling curve diagram and (**b**) heating curve diagram.

To further explore the effect of NFC addition on PLA crystallinity and melting behavior, DSC tests were conducted. Figure 5a presents cooling curves. Pure PLA shows no crystallization peak. However, with just 1 wt% NFC, a peak emerges around 100 °C. As NFC content rises, the crystallization temperature increases, suggesting that NFC promotes heterogeneous nucleation [22]. The crystallization peak area first grows (≤10 wt% NFC) and then declines (>10 wt% NFC), indicating optimal crystallization rate with moderate NFC amounts. Figure 5b depicts second heating curves (see Table 1 for details). Cold crystallization occurs due to incomplete crystallization during cooling. NFC addition sharply reduces ΔHcc, highlighting its role in enhancing PLA’s crystallization rate. Tg rises with NFC, reflecting stronger interfacial adhesion between NFC and PLA, restricting chain movement. Tcc drops, suggesting NFC’s role as a heterogeneous nucleating agent to uniform mixing of NFC and PLA. A melting peak appears around 160 °C, with pure PLA showing a double peak. This reflects a melting–recrystallization process, with peaks at 157.2 °C and 164.5 °C corresponding to defective and perfected crystals, respectively. NFC addition notably increases crystallinity (Χc), peaking at 10% NFC, aligning with XRD findings. This suggests that NFC creates more nucleation sites, promoting crystallization.

#### 3.2.4. Rheological Properties Analysis

**Figure 6:**
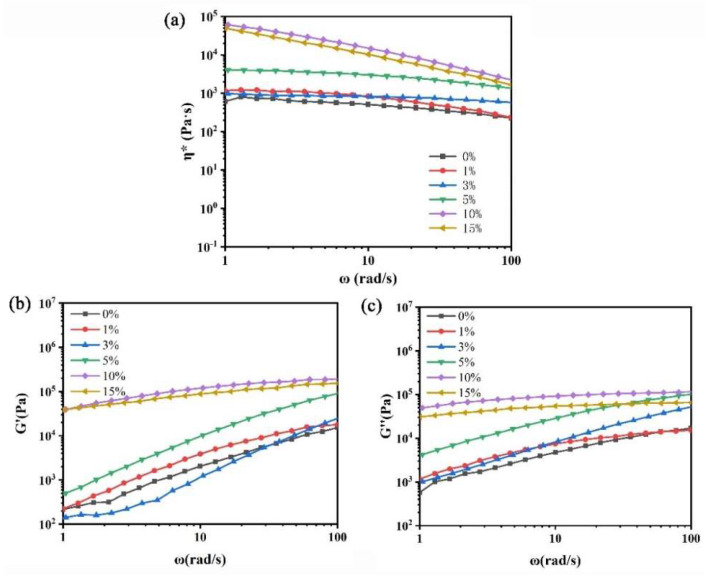
Rheological properties of PLA and PLA/NFC composites with different mixtures. (**a**) η*–ω relation curve, (**b**) G′–ω relation curve, and (**c**) G″–ω relation curve.

To further evaluate the microstructural effects of NFC in PLA and gain a deeper understanding of the processing characteristics of the prepared nanocomposites, Figure 6 illustrates the rheological properties of pure PLA and PLA/NFC nanocomposites tested at 180 °C. Evidently, the melt rheological behavior of pure PLA follows the typical pattern of molten polymers as a function of ω. The incorporation of NFC clearly influences the melt rheology of PLA, adhering to a power–law relationship, particularly at low frequencies.

From the η*–ω relationship curve in Figure 6a, it can be observed that, within the ω range of 1–100 rad/s, the η* values of both pure PLA and PLA/NFC nanocomposites decrease with increasing ω. This shear-thinning behavior is attributed to the facilitated chain disentanglement and orientation of PLA and NFC molecular chains along the shear direction as ω increases, thereby reducing viscosity. Vertically, as NFC content increases at the same ω, the material’s η* also gradually rises. When NFC content reaches 10 wt%, η* increases significantly due to the enhanced interfacial interaction between uniformly dispersed NFC and the PLA matrix. NFC hinders the free movement of PLA chains and acts as physical crosslinking points, promoting the formation of an interconnected spatial network structure between PLA chains and NFC. However, when NFC content reaches 15 wt%, aggregation occurs in the PLA matrix, reducing crosslinking points and intermolecular forces, leading to a slight decrease in η* for PLA/NFC nanocomposites.

Figure 6b shows the relationship between G′ and ω for pure PLA and PLA/NFC nanocomposites at 180 °C. As ω increases, G′ values for both pure PLA and PLA/NFC nanocomposites rise significantly. At the same ω, pure PLA has the lowest G′. With increasing NFC content, G′ gradually increases due to restricted PLA chain movement, increased physical crosslinking points, enhanced NFC–PLA interactions, and the formation of an interconnected network structure. However, at 15 wt% NFC, aggregation occurs, increasing free volume and slightly decreasing G′. Figure 6c illustrates the relationship between G″ and ω. Similar to G′, G″ values increase significantly with ω for both materials. At the same ω, pure PLA has the lowest G″. NFC incorporation enhances G″ and, as NFC content rises, G″ gradually increases due to restricted PLA chain movement, increased entanglement, and intermolecular forces. At 15 wt% NFC, aggregation occurs, reducing crosslinking, and increasing free volume, leading to a slight decrease in G″.

#### 3.2.5. TGA-FTIR Analysis

**Figure 7:**
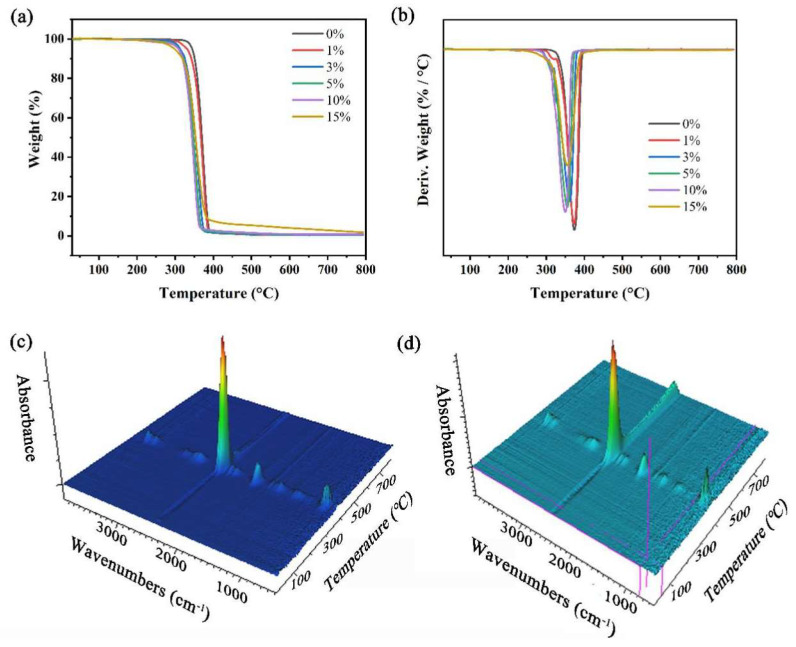
Thermogravimetric and infrared analysis. (**a**) TGA and (**b**) DTG of PLA and PLA/NFC composites with different mixture ratios, and TGA–FTIR of (**c**) PLA and (**d**) 10 wt% PLA/NFC.

To study the thermal stability and structure of PLA and PLA/NFC composites, a combined thermogravimetric and infrared analysis was conducted (Figure 7). Results, shown in Table 2, indicate that adding NFC lowers the initial and maximum decomposition temperatures (T_0_ and T_max_), likely due to NFC’s lower decomposition temperature (≈210 °C) and residual metal ions catalyzing decomposition. Despite this, T0 remains above 300 °C, suitable for most uses. Both composites and pure PLA show a single degradation peak (300–450 °C), suggesting no structural reaction between NFC and PLA and indicating good mixing. IR analysis confirms no chemical structure changes or new bonds. The main decomposition products are CO_2_ and CO.

#### 3.2.6. Mechanical Properties Analysis

**Figure 8:**
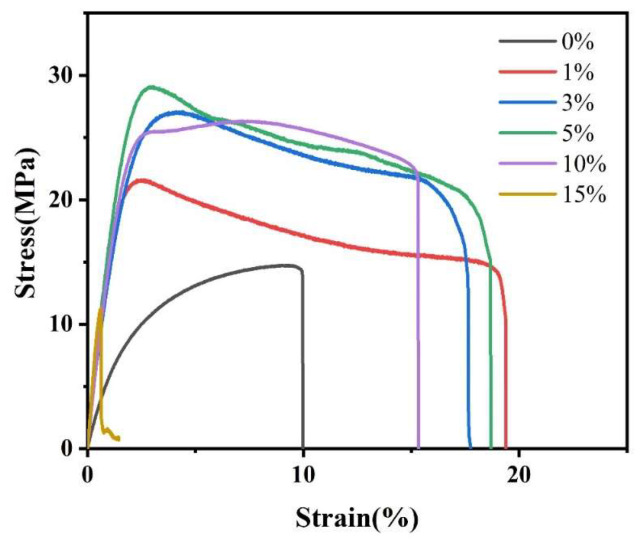
Mechanical properties curves of PLA and PLA/NFC composites with different mixed proportions.

Figure 8 displays mechanical performance curves for PLA/NFC composites with varying NFC additions, and Table 3 outlines their specific mechanical parameters. It is evident that composites with NFC exhibit higher tensile strength and strain than pure PLA, except at 15 wt% NFC due to uneven dispersion. At 1 wt% NFC, the tensile strain peaks at 19.39, an 87.9% increase from pure PLA. When NFC is at 5 wt%, tensile strength reaches a maximum of 29.26 MPa, a 98.8% boost. This underscores NFC’s significant enhancement effect on PLA. However, as NFC content rises, composite strength increases while strain decreases. This is attributed to NFC’s high tensile strength and Young’s modulus, augmenting PLA’s mechanical properties. Enhanced crystallinity due to NFC addition also contributes, as it fosters an intertwined network of molecular chains, hindering PLA movement. Notably, despite the highest crystallinity at 10 wt% NFC, tensile strength is not the highest, possibly due to surface defects. Additionally, most composites with NFC show a “plateau” curve, indicating material yielding. This suggests improved crystallinity leads to PLA molecular chain orientation, crystal fragmentation, and slip during stretching.

#### 3.2.7. Moisture Permeability Analysis

**Figure 9:**
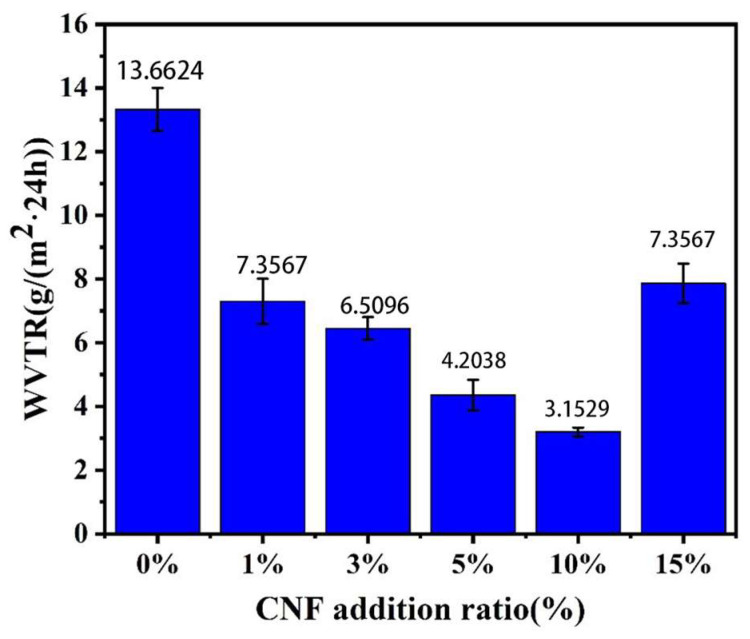
Effect of NFC addition on the water vapor transmission rate of PLA.

A material’s moisture permeability is indicated by WVTR; a lower rate signifies better performance. Figure 9 shows how NFC additions affect PLA/NFC composites’ WVTR. As the amount of CNF added gradually increased, WVTR showed a trend of first decreasing and then increasing. WVTR decreases with NFC addition up to 10 wt%, reaching a minimum of 3.1529 g/m^2^·24 h, 76.9% lower than pure PLA. This is because NFC enhances PLA crystallinity, blocking water molecules, and its high aspect ratio creates a tortuous path in PLA, reducing vapor permeation. However, at 15 wt% NFC, WVTR rises due to NFC aggregation, decreasing crystallinity and water vapor barrier.

## 4. Conclusions

In this study, PLA/NFC composite films were successfully prepared. The interfacial compatibility of cellulose was effectively modified to improve the mechanical properties and water resistance of the material. The stability of the composites and the enhancement of PLA by NFC were reflected by various performance tests. We solved the sore spots of PLA usage and highlighted the potential of PLA/NFC in packaging applications. Given the significant enhancements in mechanical and barrier properties observed in PLA/NFC composites, these materials are potential candidates for applications in food packaging, biomedical materials, and other fields. It is essential to evaluate their effectiveness and feasibility in practical applications, which will facilitate continued in-depth research into the biodegradability and environmental friendliness of PLA/NFC composite materials, thereby contributing to sustainable development and environmental protection.

## Figures and Tables

**Table 1:** DSC parameters of PLA and PLA/NFC composites during the second heating process.

Samples	T_g_ (°C)	T_cc_ (°C)	T_m1_ (°C)	T_m2_ (°C)	ΔH_cc_ (J/g)	ΔH_m_ (J/g)	Χ_c_ (%)
0 wt%	54.3	108	157.2	164.5	24.573	32.691	8.67
1 wt%	55.3	96.4	-	163	6.1645	33.977	30.01
3 wt%	55.2	94.6	-	163.2	5.7836	34.749	31.90
5 wt%	54.3	97	-	163.3	5.9094	34.697	32.37
10 wt%	55.3	98.2	-	163.3	4.6607	33.768	34.55
15 wt%	54.7	99.1	-	163.5	6.9554	28.841	27.51

Note: Tg indicates the starting temperature, Tcc and Tm both indicate the peak temperature.

**Table 2:** Thermal degradation parameters of PLA/NFC composites with different amounts of NFC.

Samples	TG		
	T_0_ (°C)	T_max_ (°C)	Residual Rate (%)
0 wt%	345.2	373.4	0.2
1 wt%	331.3	370.6	0.43
3 wt%	313.0	361.4	0.55
5 wt%	309.1	355.7	0.82
10 wt%	309.1	350.0	0.96
15 wt%	360.6	354.4	1.82
standard deviation values	19.62	8.55	0.52

**Table 3:** Mechanical property parameters of PLA and PLA/NFC composites.

Samples	Stress (MPa)	Strain (%)
0 wt%	14.72	10.32
1 wt%	22.35	19.39
3 wt%	27.08	17.95
5 wt%	29.26	18.67
10 wt%	26.30	15.71
15 wt%	-	-

## Data Availability

No new data were created or analyzed in this study. Data sharing is not applicable to this article.
